# Synthesis, Anticancer Activity and UPLC Analysis of the Stability of Some New Benzimidazole-4,7-dione Derivatives

**DOI:** 10.3390/molecules19010400

**Published:** 2013-12-31

**Authors:** Katarzyna Błaszczak-Świątkiewicz, Diogo Correia Almeida, Maria De Jesus Perry, Elżbieta Mikiciuk-Olasik

**Affiliations:** 1 Department of Pharmaceutical Chemistry and Drug Analysis, Medical University, Muszyńskiego 1, Łódź 90-151, Poland; E-Mail: elzbieta.mikiciuk-olasik@umed.lodz.pl; 2 Department of Pharmaceutical Chemistry and Therapeutics, Faculty of Pharmacy, University of Lisbon, Av. Prof. Gama Pinto, Lisboa 1649-003, Portugal; E-Mails: diogo.correia.almeida@gmail.com (D.C.A.); mjprocha@ff.ul.pt (M.D.J.P.)

**Keywords:** anticancer prodrug, hypoxia, benzimidazole-4,7-dione, *N*-oxide benzimidazole-4,7-dione, UPLC

## Abstract

In this work, a sensitive analytical method to study the stability of two new series of synthesized heterocyclic compounds, the benzimidazole-4,7-diones **5** and *N*-oxide benzimidazole-4,7-dione derivatives **6** was established and validated. These derivatives were developed as potential anticancer substances to be activated under hypoxic conditions. At this point we were concerned with establishing their stability in some specific environments for further biological studies. For that, we developed and validated an RP-UPLC method. Next, selected compounds were tested *in vitro* for possible anticancer activity. Their effect on A549 tumour cell lines under normoxia and hypoxia conditions was determined by a WST-1 test. Four of the examined compounds (compounds **5a**–**c** and **6c**) showed very good antiproliferative effects and three of them (compounds **6a**, **6b** and **6d**) were specific for hypoxia conditions. The hypoxia/normoxia cytotoxic coefficient of compound **6b** is close to that of tirapazamine—a reference compound in our experiments—and this parameter locates it between mitomycin C and 2-nitroimidazole (misonidazole).

## 1. Introduction

**Figure 1:**
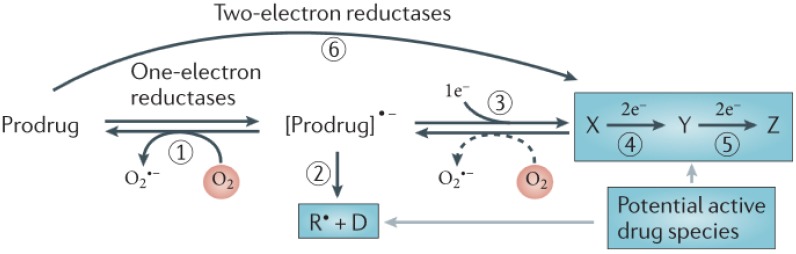
One-electron *versus* two-electron reduction of bioreductive prodrug [2].

Cancer is still one of the leading causes of death in the World, particularly in developing countries. Hypoxia is present in most of those tumours and its characteristic feature is the variable incidence and severity within a given patient population, which happens because solid tumour tissue is poorly perfused. There is evidence that tumours exhibiting extensive hypoxia are more aggressive and, therefore, they have a poorer prognosis, since hypoxia is implicated in radiotherapeutic and chemotherapeutic resistance. Currently there is a wide range of drugs, mainly hypoxic-activated bioreductive prodrugs, that attempt to address this issue, tirapazamine being the most representative molecule of such prodrugs. A prodrug is defined as an inactive compound that is converted in the body, either spontaneously or through metabolism by some endogenous enzymes, into the desired, pharmacologically active species [1]. Bioreductive prodrugs are converted in hypoxic areas into cytotoxic species by enzymatic reduction [2,3]. Nitroaromatic groups, quinones, aromatic *N*-oxides and aliphatic *N*-oxides are some of the chemical groups which have the potential to be metabolised by enzymatic reduction under hypoxic conditions, and thus provide the basis for the design of bioreductive prodrugs [2]. The most common mechanism by which bioreductive prodrugs are selective for hypoxic cells involves one-electron reduction which generates a prodrug radical that can be re-oxidized by oxygen (reaction 1 in Figure 1) in normal cells (forming superoxide), but generates an active drug in hypoxic cells, either by fragmentation of the prodrug radical (reaction 2 in Figure 1) or by its further reduction, usually by disproportionation (reaction 3 in Figure 1) and subsequent reduction of the two electron reduction of product X, (reactions 4 and 5 in Figure 1). This is characteristic of nitro and aromatic *N*-oxide compounds and results in hypoxia-selective cell killing provided that the prodrug radical (or its downstream products) are more cytotoxic than superoxide or the unreduced prodrug [2,3].

The second mechanism, typical of quinones, includes bioreductive activation with two sequential one-electron reductions [2]. Nitroaromatic compounds, such as CB1954, are hypoxia-activated prodrugs. The one-electron adduct (the nitro radical anion) can be scavenged by molecular oxygen, restricting activation in hypoxic cells [3,4,5]. The aromatic *N*-oxide class is best represented by tirapazamine (TPZ), which is the clinically most useful hypoxia-activated prodrug discovered to date. TPZ shows a high hypoxic selectivity (100 to 200 fold) in cell suspension cultures, but its diffusion through tissue is limited by its metabolism into the non-diffusible, radical species. Nevertheless, this drug is selectively toxic to the hypoxic fraction of cells in several animal tumours *in vivo*. This specific hypoxic cytotoxicity results from the fact that the TPZ radical is much more cytotoxic than the superoxide radical [3,6,7]. It has been suggested that TPZ has a dual mechanism of action, both generating DNA radicals and, also oxidizing these radicals to form DNA breaks [8]. It was shown that TPZ enhances the antitumor efficacy of some other antitumor drugs, namely cisplatin [9,10,11,12,13,14]. Derivatives containing benzimidazole rings are active as human DNA topoisomerase I inhibitors [15,16,17] and are expected to possess anticancer properties [18] and selective affinity for cells under hypoxic conditions [15]. In this context, benzimidazole-4,7-dione derivatives play an important role, as quinones are known to have a noticeable antitumor activity [19,20,21,22,23].

**Figure 2:**
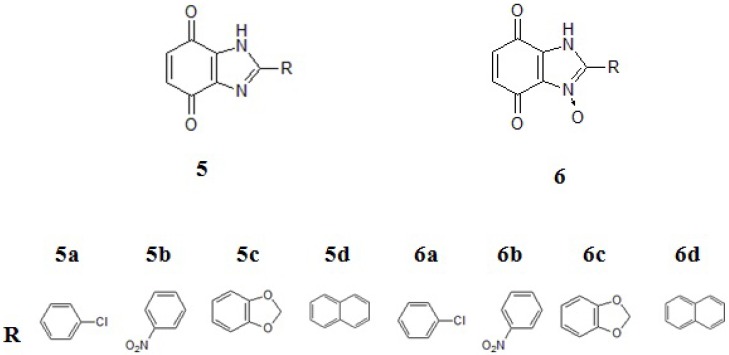
Structural formula of benzimidazole-4,7-dione derivatives. **a**: *p*-chlorophenyl, **b**: *o*-nitrophenyl, **c**: piperonyl, **d**: naphthyl.

These compounds are being worked on intensively as they might have new potential anticancer properties [24,25,26,27,28]. This was the reason for initiating our experiments with a group of new benzimidazole-4,7-dione derivatives **5** and their analogues 6 with *N*-oxide bonds (Figure 2).

The main aim of this study was to describe the method of synthesis to obtain derivatives **5** and **6**, evaluate their stability in different environments by UPLC analysis, and also quantify their biological activities under normoxic and hypoxic conditions.

## 2. Results and Discussion

### 2.1. Chemistry

**Scheme 1:**
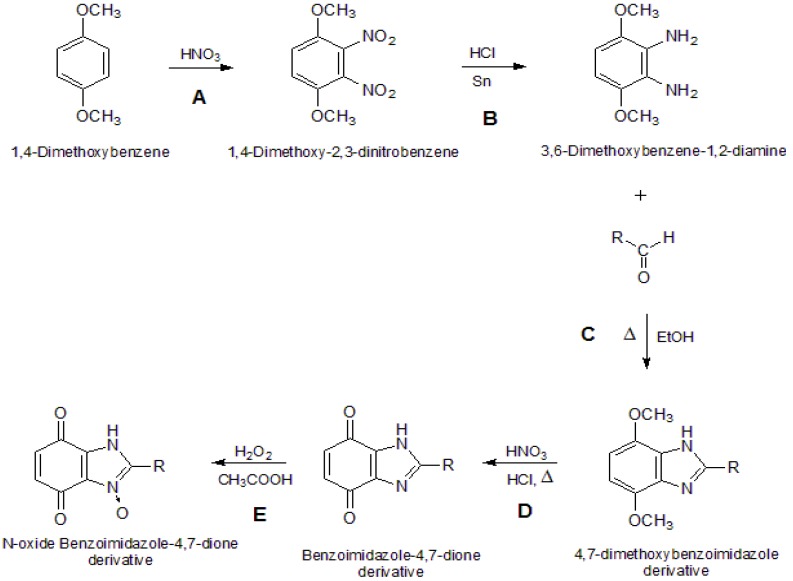
General synthesis reaction.

The syntheses of the benzimidazole-4,7-dione derivatives **5** compounds and the *N*-oxide benzimidazole-4,7-dione derivatives **6** comprises a series of known reactions (Scheme 1). Reactions **A** (aromatic nitration) and **B** (reduction of nitro compounds) are common to all products. Reaction **C** was adapted from the literature and comprises the reaction between diamine **3** and different aldehydes to produce benzimidazole-4,7-dione derivatives **4** [15]. They were obtained by the direct cyclocondensation of a suitable diamine with a suitable aldehyde in an anhydrous solvent at its boiling point [15]. Reaction **D** is an oxidation of product **4**, and it was accomplished using oxidizing agents such as nitric acid, which works well for highly substituted 1,4-dimethoxybenzene derivatives and requires strongly acidic media [29]. Reaction **E** was performed to produce the *N*-oxide benzimidazole-4,7-dione derivatives, which were obtained by the direct reaction of 30% solution of hydrogen peroxide in glacial acetic acid with the previously obtained benzimidazole-4,7-dione derivatives [15]. The benzimidazole ring structure of the main product of cyclocondensation of a diamine with an aldehyde was established by X-ray crystal structure analysis [15].

### 2.2. UPLC Analysis

#### 2.2.1. Optimisation

Using an isocratic system consisting of a mixture of acetonitrile and ammonium formate buffer, the retention times for compounds **5b** and **6b** were 4.3 and 3.9 min, respectively (Table 1). This valuable difference may be due to the protonation of the Schiff base without the *N*-oxide bond. This does not occur with the *N*-oxide derivative. This theory is supported by the chromatogram (Supplementary Material Figure S1) of product **5b**, which presents its minimum and the maximum absorbance for the protonated and non-protonated forms, respectively. The chromatogram of **6b** only shows the maximum absorbance for the non-protonated form. The use of this mobile phase at a flow rate of 0.5 mL/min was found to be optimum and provided adequate peak separation and resulted in the best resolution among all the other combinations tested.

**Table 1:** The obtained values of Rt, k, n for the isocratic system acetonitrile/ammonium formate buffer (30:70 v/v, pH = 2.4).

Concetration 10^−1^ mg/mL	Rt_mean_ (min)	k_mean_	n_mean_
5b	4.3	1.24e^1	7.74e^3
6b	3.9	1.20e^1	6.31e^3

Rt, retention time; k, retention coefficient; n, number of theoretical plates.

#### 2.2.2. Validation

*Precision*: To determine the compound content in the sample, a calibration curve was generated for each compound as described earlier. The following parameters: arithmetic mean (*x*), standard deviation (s), mean ± SD (µ), and relative standard deviation [RSD (%)] were used to define the precision of the method for each compound. The results are presented in Supplementary Material Table S1).

*Accuracy*: To determine the compound content in the sample, a calibration curve was generated for each compound as described earlier. A degree of recovery was defined for each substance content expressed in mg/mL and the parameters: *x*, s, µ, RSD (%) were used to determine the accuracy of the method for each compound. The results are presented in Supplementary Material Table S2).

*Linearity*: A linear relationship between the particular sample concentration and peak area was drawn. Equations (1) and (2) represent the calibration curves for compounds **5b** and **6b**, respectively. Correlation coefficients were also determined:

y = 6.54 × 10^6^*x* − 3.39 × 10^3^ R^2^ = 0.9999 (1)

y = 9.10 × 10^6^*x* − 1.41 × 10^4^ R^2^ = 0.9998 (2)

*LOD*: This parameter was calculated as an arithmetical mean from three individual measurements. The limit of detection, at a signal-to-noise ratio of 3, were 0.304 µg/mL and 0.849 µg/mL for compounds **5b** and **6b**, respectively.

*LOQ*: This parameter was calculated as an arithmetical mean from three individual measurements. The limit of detection, at a signal-to-noise ratio of 10, were 1.01 µg/mL and 2.83 µg/mL for compounds **5b** and **6b**, respectively.

The accuracy and precision of the method were within the acceptable limits of ±5%.

#### 2.2.3. Stability of Compounds in Different Environments

The purpose of this analysis was to assess the media in which compounds **5b** and **6b** are more stable, so that it can be used in further biological studies. For this purpose, it is necessary for the compounds to be as stable as possible in these media, *i.e.*, possessing a high t_1/2_. The percentage of sample recovery in each environment is presented in Table 2. The results of the degradation process for compounds **5b** and **6b** (t_1/2_ and degradation rate) are summarized in Table 3.

Both compounds seem to be rather stable in all solvents and within a time of up to 72 h do not undergo relevant decomposition. Apparently, compound **5b** did not suffer any degradation in water and methanol. This fact is proved by the high mean recovery in these solvents (Table 2). In theory, all of these environments can be used in further biological studies, and the choice falls on the most suitable one.

**Table 2:** Sample recovery (arithmetic mean) after 72 h in the specific environment.

**Compound 5b**	
Solvent	Recovery (%)
0.9% NaCl	77.0
H_2_O	100.0
0.2% DMSO	83.0
MeOH	98.2
**Compound 6b**	
0.9% NaCl	78.2
H_2_O	77.9
0.2% DMSO	90.4
MeOH	76.1

DMSO, dimethylsulfoxide; MeOH, methanol.

**Table 3:** Degradation kinetic parameters in tested solvents.

**Compound 5b**			
Solvent	*k* (h^−1^)	t_1/2_ (h)	R^2^
0.9% NaCl	0.0004	155	0.98
H_2_O	^a^	^a^	^a^
0.2% DMSO	0.0002	215	0.93
MeOH	^a^	^a^	^a^
	**Compound 6b**		
0.9% NaCl	0.0004	142	0.97
H_2_O	0.0004	146	0.86
0.2% DMSO	0.00009	413	0.99
MeOH	0.0002	161	0.99

^a^ No degradation.

### 2.3. Biological Activities under Normoxia and Hypoxia Conditions

The anticancer activity of newly synthesized benzimidazole derivatives **5** and *N*-oxide benzimidazole derivatives **6** was investigated *in vitro* on human lung adenocarcinoma A549 cells. The results were expressed as a fraction of viable cells. Changes in cell morphology induced by the compounds were visualized with a phase-contrast microscope. Tirapazamine was used as a reference drug.

#### 2.3.1. Cytotoxicity at Normoxia

The concentration-response analysis was performed to determine the compound concentrations required to inhibit the growth of cancer cells by 50% (IC_50_) after an incubation of 48 h. The synthesized compounds were tested in a wide range of concentrations, from 1 to 500 μM. Four out of eight tested compounds (compounds **5b** and **5d**, **6b** and **6d**) showed IC_50_ values less than 100 μM in cells cultured under normoxic conditions (Table 4). In comparison, under the same experimental conditions 166 μM tirapazamine was required to inhibit the growth of A549 cells by 50%. Compounds **5a**, **5c** and **6c** were found to have a higher anticancer activity, with an IC_50_ value of 30.2 ± 1.2 μM, 80.9 ± 1.9 μM and 79.5 ± 1.9, respectively. The culture of A459 cells in the presence of compound **5a** at 10 µM caused approximately 33% inhibition of the cell growth compared to control cells. However, the treatment of cells with compound **6a** at the same concentration decreased cell viability by 20%. These results demonstrated a much higher effectiveness of compound **5a** compared to compound **6a** at low concentration (10 µM). A smaller effect on the cell growth was observed after the treatment with benzimidazole-4,7-dione derivatives **5b** and **6a** (IC_50_ values of 100.0 ± 1.8 μM and 115.7 ± 1.9 μM, respectively). Other tested benzimidazole-4,7-diones (compounds **5b**–**d** and **6b**–**d**) were less effective and showed a much higher value of IC_50_, over the range 252–500 μM (Table 4).

**Table 4:** *In vitro* growth inhibition of selected tumor cell lines by new benzimidazole-4,7-dione derivatives.

Compound	IC_50_ [μM] A549		Differential cytotoxicity O/H
	Normoxia (O)	Hypoxia (H)	
**5a**	30.2 ± 1.2	36.1 ± 1.2	0.83
**5b**	100.0 ± 1.8	47.4 ± 1.1	2.13
**5c**	80.9 ± 1.9	51.2±1.5	1.58
**5d**	479.5 ± 3.6	232.4 ± 1.4	2.06
**6a**	115.7 ± 1.9	35.0 ± 1.6	3.28
**6b**	500.6 ± 1.2	116.0 ± 0.8	4.31
**6c**	79.5 ± 1.9	44.0 ± 2.5	1.80
**6d**	252 ± 2.5	96.8 ± 1.9	2.62
**Tirapazamine**	166.2 ± 1.6	30.0 ± 1.5	4.61

WST-1 assay was used to determine the inhibition of the cell growth after 48 h incubation with the tested compounds. IC_50_ values (concentration of the tested compounds causing 50% inhibition of the cell growth compared to control cells) were calculated and expressed as mean ± SD, n = 3.

Our conclusions from the results are that benzimidazole derivatives which have a chlorophenyl (compounds **5a**, **6a**) or nitrophenyl substituent (compounds **5b**, **6b**) inhibited the growth of A549 cells more potently than the analogous benzimidazole-4,7-dione derivatives with piperonyl (compounds **5c**, **6c**) or naphthyl (compounds **5d**, **6d**) groups.

Comparing these results with our earlier study with other benzimidazoles, we found that both groups—benzimidazole derivatives and benzimidazole-4,7-dione derivatives—possess a very similar cytotoxic activity against the selected tumor cell lines [15,30,31].

#### 2.3.2. Cytotoxicity at Hypoxia

The tumour microenvironment characterized by hypoxia is the target of novel potential anticancer substances which have a bioreductive mechanism of action. For these reasons we also evaluated the effects of our compounds on hypoxic cancer cells. A549 cells were exposed to hypoxia (1% O_2_) for 24 h before treatment and were maintained under hypoxic conditions during culture in the presence of the compounds. As shown in Table 4, the most active agents among series **5** and **6** under hypoxic conditions were compounds **5a** and **6a** (IC_50_ values of 36.1 ± 1.2 μM and IC_50_ value 35.0 ± 1.6 μM, respectively, Supplementary Material Figure S2). Under hypoxic conditions, the cell viability was significantly decreased when cells were cultured in the presence of compounds **5b** and **6b** (IC_50_ values of 47.4 ± 1.1 μM and IC_50_ value 116.0 ± 0.8 μM, respectively). Moreover, compound **6b** was more effective in hypoxic cells compared to normoxic cells with IC_50_ values of 116.0 ± 0.8 μM and 500.6 ± 1.2 μM, respectively. The same activity is characteristic for compound **6a** (Supplementary Material Figure S2).

## 3. Experimental

### 3.1. Materials and Instrumentation

IR spectra (KBr discs) were recorded using a Mattson Infinity Series FT-IR spectrophotometer. ^1^H and ^13^C spectra were recorded on a 300 MHz Varian Mercury spectrometer (Darmstadt, Germany) in DMSO-*d*_6_ or CDCl_3_-*d*_6_ as solvents and tetramethylsilane (TMS) as internal reference. MS spectra (FAB method, M+1, M-1, matrix-glycerin) were recorded on a Finnigan Mat 95 spectrometer, Bremen, Germany). Carbon, hydrogen and nitrogen elemental analyses were performed using a Perkin Elmer 2400 series II CHNS/O (Madison, WI, USA), and agreed with the proposed structures within ± 0.3% of theoretical values.

Chromatographic purification was performed on silica gel plates (Merck F_254_, Darmstadt, Germany) with the indicated eluents. Chemicals and solvents were obtained from commercial sources. The UPLC system consisted of an Acquity Ultra Performance LC Waters H-class system (Waters, Corporation, Milford, MA, USA). Chromatographic separation was achieved on an Acquity UPLC HSS T3 column (1.8 µm × 2.1 × 50 mm). The chromatographic peaks were identified with the Waters PDA eλ Acquity UPLC Detector. Compound **5b** was monitored at a wavelength of 261 nm and compound **6b** at 289 nm. All chromatographic experiments were performed in an isocratic mode. The mobile phase consisted of a mixture of solvents of chromatographic purity, acetonitrile/buffer (30:70 v/v). Buffer was a 0.01 M mixture of ammonium formate and formic acid pH = 2.4. The flow rate was set to 0.5 mL/min and the oven temperature to 25 °C. Compound samples were weighed on analytical scales with an accuracy of 0.01 mg. All the samples were prepared in 5, 10 and 25 mL volumetric flasks. Solution volume injections of 1.5 µL were performed with the use of an autosampler. Each sample was analysed three times and the run time analysis was five minutes. All solutions were filtered through a 0.45 µm membrane filter. The final result was presented as an arithmetical mean. The control of the UPLC system and detection was achieved by the Empower 3 Waters^®^ software. Solvents for UPLC analysis were of UPLC grade and obtained from J. T. Baker (Center Valley, PA, USA). The method was validated in terms of precision, accuracy, specificity, detectability, and stability for compounds **5b** and **6b**.

The human lung adenocarcinoma A549 cell line, purchased from Health Protection Agency Culture Collections (ECACC, Salisbury, UK), was cultured in F12K medium (HyClone, Loughborough, UK) supplemented with 10% heat-inactivated foetal bovine serum (FBS), penicillin (10 U/mL) and streptomycin (10 µg/mL) in air with 5% CO_2_ at 37 °C. Hypoxic cells were obtained by culturing in a hypoxic incubator in 1% O_2_ and 5% CO_2_ at 37 °C for 24 h before treatment.

In order to determine the anticancer activity of the analysed compounds we evaluated the cell viability using THE WST-1 assay (Millipore, Billerica, MA, USA) according to the manufacturer’s instructions.

The absorbance was determined at 420 nm using a microplate reader (Synergy H1, Bio-Tek, Winooski, VT, USA) after 3 h of incubation at 37 °C. IC_50_ values (concentration of the tested compounds required to reduce cell density to 50%) were calculated by the concentration-response curve fitting using a Microsoft Excel-based analytic method.

### 3.2. Methods

#### 3.2.1. Chemistry

The synthesis of compounds **2** and **3** are well known reactions [32]. The chemical reaction to produce derivatives **4** was described by the authors in another paper [15].

*Synthesis of 1H-benzimidazole-4,7-dione Derivatives* **5** [31]. The 4,7-dimethoxybenzimidazole derivative **4** (2.89 g; 10 mmol) was suspended in HCl 30% (15 mL). The suspension was heated under reflux at 80–90 °C, with constant stirring. Next, HNO_3_ 90% (5 mL), was added dropwise over one hour. The mixture was stirred at room temperature for 24 h and the crude precipitate was filtered off. The final product was recrystallized from ethanol. This synthesis was repeated in the same way, individually, to give products **5a**–**d**.

*2-(4-Chlorophenyl)-1H-benzimidazol-4,7-dione* (**5a**). Yield 65%, IR (KBr) υ/cm^−1^: 3615 (NH), 1690 (C=O), 1485 (C=N); ^1^H-NMR (DMSO-*d*_6_) δ: 14.5 (s,1H,NH), 8.2 (d, 2H, CH, *J* = 2.0 Hz), 7.6 (d, 2H, CH, *J* = 2.0 Hz), 7.5 (d, 2H, CH, *J* = 7.9); ^13^C-NMR (DMSO-*d*_6_) δ: 180.0, 179.1, 150.2, 143.4, 142.8, 136.8, 135.7, 129.9, 129.3, 128.7, 127.9; MS *m/z* [M+1, M−1]: 259, 257; calculated for C_13_H_7_ClN_2_O_2_: C 60.36, H 2.73, N 10.83; found: C 60.43, H 2.80, N 10.60. R_f_ (chloroform/methanol—6.25% v/v) = 0.49.

*2-(2-Nitrophenyl)-1H-benzimidazol-4,7-dione* (**5b**). Yield 45%, IR (KBr) υ/cm^−1^: 3391 (NH), 1690 (C=O), 1526 (NO_2_asym), 1347 (NO_2_sym), 1481 (C=N); ^1^H-NMR (DMSO-*d*_6_) δ: 9.2 (s, 1H, NH), 8.1 (m, 4H, CH), 7.8 (d, 2H, CH, *J* = 7.9 Hz); ^13^C-NMR (DMSO-*d*6) δ: 181.2, 179.1, 148.7, 147.2, 146.7, 146.1, 142.9, 141.9, 133.1, 131.9, 128.1, 125.5, 124.6: MS *m/z* [M+1, M−1]: 270, 268; calculated for C_13_H_7_N_3_O_4_: C 58.00, H 2.62, N 15.61; found: C 58.12, H 2.64, N 15.67. R_f_ (chloroform/methanol—6.25% v/v) = 0.51.

*2-Benzo[1,3]dioxol-1H-benzimidazol-4,7-dione* (**5c**). Yield 65%, IR (KBr) υ/cm^−1^: 3324 (NH), 2960 (CH_2_), 1701 (C=O), 1503 (C=N), 1264 (C-O-Csym), 1036 (C-O-Casym); ^1^H-NMR (DMSO-*d*_6_) δ: 4.0 (s, 1H, NH), 7.9 (d, 2H, CH, *J* = 8.0 Hz), 7.2 (d, 1H, CH, *J* = 7.9 Hz), 7.0 (d, 2H, CH, *J* = 1.98 Hz), 6.2 (s, 2H, CH_2_); ^13^C-NMR (DMSO-*d*_6_) δ: 179.5, 179.0, 149.1, 148.6, 147.6, 142.1, 141.5, 141.3, 141.1, 129.1, 120.3, 115,1, 113.2, 91; MS *m/z* [M+1, M−1]: 269, 267; calculated for C_14_H_8_N_2_O_4_:C 62.69, H 3.01, N 10.44; found: C 62.55,H 2.99, N 10.50. R_f_ (chloroform/methanol—6.25% v/v) = 0.50.

*2-Naphthyl-1H-benzimidazol-4,7-dione* (**5d**). Yield 55%, IR (KBr) υ/cm^−1^: 3424 (NH), 3033 (ArH), 1677 (C=O ); 1452 (C=N); ^1^H-NMR (DMSO-*d*_6_) δ: 13.0 (s,1H,NH), 8.7 (s,1H,CH) 8.3 (d, 2H, CH, *J* = 1.8 Hz), 8.1 (d, 2H, CH, *J* = 1.8 Hz), 7.9 (d, 2H, CH, *J* = 1.8 Hz), 7.6 (d, 2H, CH, *J* = 8.1 Hz); ^13^C-NMR (DMSO-*d*_6_) δ: 179.9, 179.1, 150.2, 148.6, 148.5, 147.7, 147.3, 142.2, 141.2, 134.1, 133.8, 133.6, 128.5, 126.5, 124.3; MS *m/z* [M+1, M−1]: 275, 273; calculated for C_17_H_10_N_2_O_2_: C 74.44, H 3.67,N 10.21; found: C 74.30, H 3.62, N 10.47. R_f_ (chloroform/methanol—6.25% v/v) = 0.50.

*Synthesis of N-oxide 1H-benzimidazole-4,7-dione derivatives* **6** [15]. The benzimidazole-4,7-dione derivative **5** (2.59 g; 10 mmol) was mixed with anhydrous acetic acid (15 mL). Next, one first portion of hydrogen peroxide (5 mmol) was added. The mixture was heated under reflux at 50–60 °C. After six hours, the second equal portion of hydrogen peroxide was added. After 24 h heating the mixture was concentrated in vacuum to a small volume, diluted with methylene chloride and washed with sodium carbonate solution. The organic layer was dried, concentrated in vacuum and diluted with diethyl ether. The solid precipitate was filtered off and recrystallized from isopropanol. This synthesis was made in the same way, individually, to compounds **5a**–**d** to give products **6a**–**d**, respectively. The chromatographic purity of the obtained compounds was confirmed by TLC as above.

*2-(4-Chlorophenyl)-1H-benzimidazol-4,7-dione N-oxide* (**6a**). Yield 50%, IR (KBr) υ/cm^−1^: 3445 (NH), 1685 (C=O), 1661 (C=O), 1484 (C=N), 1282 (N-O); ^1^H-NMR (DMSO-*d*_6_) δ: 14.5 (s, 1H, NH), 8.1 (d, 2H, CH, *J* = 2.0 Hz), 7.6 (d, 2H, CH, *J* = 2.0 Hz), 7.5 (d, 2H, CH, *J* = 8.0 Hz); ^13^C-NMR (DMSO-*d*_6_) δ: 180.2, 179.3, 157.2, 143.1, 142.6, 136.5, 135.2, 129.8, 129.1, 128.6, 128.0; MS *m/z* [M+1, M−1]: 275, 273; calculated for C_13_H_7_ClN_2_O_3_: C 56.85, H 2.57, N 10.20; found: C 56.53, H 2.70, N 10.45. R_f_ (chloroform/methanol—6.25% v/v) = 0.50.

*2-(2-Nitrophenyl)-1H-benzimidazol-4,7-dione N-oxide* (**6b**). Yield 75%, IR (KBr) υ/cm^−1^: 3480 (NH), 1682 (C=O), 1541 (NO_2asym_), 1347 (NO_2sym_) 1434 (C=N), 1251 (N-O); ^1^H-NMR (DMSO-*d*_6_) δ: 10 (s, 1H, NH), 8.2 (m, 4H, CH), 7.9 (d, 2H, CH, *J* = 7.9 Hz); ^13^C-NMR (DMSO-*d*_6_) δ: 180.2, 179.3, 157.7, 147.6, 146.2, 146.0, 143.0, 142.4, 133.5, 131.5, 128.4, 125.3, 124.1: MS *m/z* [M+1, M−1]: 286, 284; calculated for C_13_H_7_N_3_O_5_: C 54.74, H 2.47, N 14.73; found: C 54.92, H 2.22, N 15.02. R_f_ (chloroform/methanol—6.25% v/v) = 0.50.

*2-Benzo[1,3]dioxol-1H-benzimidazol-4,7-dione N-oxide* (**6c**). Yield 35%, IR (KBr) υ/cm^−1^: 3346 (NH), 2961 (CH_2_), 1680 (C=O), 1470 (C=N), 1255 (C-O-C_sym_), 1045 (C-O-C_asym_), 1249 (N-O); ^1^H-NMR (DMSO-*d*_6_) δ: 10.0 (s, 1H, NH), 7.8 (d, 2H, CH, *J* = 8.0 Hz), 7.7 (d, 1H, CH, *J* = 8.0 Hz), 7.6 (d, 2H, CH, *J* = 2.0 Hz), 6.0 (s, 2H, CH_2_); ^13^C-NMR (DMSO-*d*_6_) δ: 18025, 179.8, 159.1, 148.2, 147.1, 142.0, 141.7, 141.5, 141.0, 129.3, 120.1, 115.5, 113.3, 91.2; MS *m/z* [M+1, M−1]: 285, 283; calculated for C_14_H_8_N_2_O_5_: C 59.16, H 2.84, N 9.86; found: C 59.11, H 2.89, N 9.80. R_f_ (chloroform/methanol—6.25% v/v) = 0.50

*2-Naphthyl-1H-benzimidazol-4,7-dione N-oxide* (**6d**). Yield 70%, IR (KBr) υ/cm^−1^: 3300 (NH), 3031 (ArH), 1670 (C=O), 1465 (C=N), 1261 (N-O); ^1^H-NMR (DMSO-*d*_6_) δ: 13.1 (s, 1H, NH), 8.8 (s, 1H, CH), 8.3 (d, 2H, CH, *J* = 2.0 Hz), 8.0 (d, 2H, CH, *J* = 2.0 Hz), 7.6 (d, 2H, CH, *J* = 2.0 Hz), 7.4 (d, 2H, CH, *J* = 7.9 Hz); ^13^C-NMR (DMSO-*d*_6_) δ: 179.5, 179.2, 160.1, 147.7, 147.5, 142.6, 142.1, 141.0, 140.2, 134.3, 133.7, 133.5, 128.1, 126.2, 124.7; MS *m/z* [M+1, M−1]: 291, 289; calculated for C_17_H_10_N_2_O_3_: C 70.34, H 3.47, N 9.65; found: C 70.30, H 3.62, N 10.17. R_f_ (chloroform/methanol—6.25% v/v) = 0.50

#### 3.2.2. UPLC Analysis

##### 3.2.2.1. Optimisation

The optimisation of the analytical system allowed determining the separation parameters for compounds **5b** and **6b**. The feasibility of various combinations of solvents such as acetonitrile and methanol in an isocratic system with altered flow rates was investigated for the complete chromatographic resolution of the compounds with the best sensitivity, efficiency, and peak shape.

##### 3.2.2.2. Validation and Evaluation Procedures of the Compounds Stability in Different Media

*Precision*: Solutions were prepared containing from 25% to 100% (v/v) of the studied compound.

*Accuracy*: Solutions were prepared containing from 25% to 100% (v/v) of the studied compound.

*Linearity*: Solutions of compound **5b** at concentrations of 0.104, 0.052, 0.0104 mg/mL, 0.0052 and 0.00104 mg/mL and solutions of compound **6b** at concentrations of 0.104, 0.052, 0.0104 and 0.0052 mg/mL were prepared. The calibration curve was obtained by plotting the peak area against the amount of the analysed substance at those concentrations, and studied by fitting the results by linear least-squares regression.

*Limit of Detection (LOD)*: Solutions of each compound with a concentration of 0.1 µg/mL were prepared.

*Limit of Quantification (LOQ)*: Solutions of each compound with a concentration of 0.1 µg/mL were prepared.

*Stability*: Each medium (1 mL, 0.2% DMSO, water, 0.9% NaCl, and HCl pH = 1.5) was added to 10 mL volumetric flasks containing products **5b** and **6b** (1 mg). The flasks were filled up with the mobile phase. Each solution was analysed at t_0_ and after 6, 24, 48, and 72 h. The obtained calibration curve was used for the determination of degradation kinetics of the compounds in the solvents. The degradation kinetic parameters: rate constant (*k*) and t_1/2_ (half-life) were calculated with the use of Equation (3), where *c_0_* is the concentration at time zero, *c* is the remaining concentration, *k* is the rate constant (h^−1^) and *t* is the time (h) [30]:


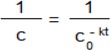
(3)

#### 3.2.3. Cytotoxicity

In order to determine the anticancer activity of the analysed compounds we evaluated the cell viability using the WST-1 assay, which is based on the conversion of the tetrazolium salt WST-1 to formazan by cellular mitochondrial dehydrogenases. Therefore, the amount of formazan dye formed directly correlates with the number of live cells in the culture.

A549 cells were seeded in 96-well plates at a density of 5,000 cells/well and cultured under normoxic conditions. To investigate the effect of compounds on hypoxic cancer cells, A549 cells were exposed to hypoxia (1% O_2_) for 24 h before treatment. Stock solutions of the tested compounds was prepared in DMSO and diluted in complete medium to give final concentrations in the range from 1 to 500 µM. Normoxic and hypoxic cells were treated with different concentrations of the tested compounds or vehicle (0.2% DMSO) for control cells. The cell viability was assessed after a 48 h incubation under normoxic or hypoxic conditions with the compounds. Briefly, WST-1 reagent was added to the cells and the percentage (%) of cell viability related to control cells was calculated by the expression [A] test/[A] control × 100 where [A] test is the absorbance of the cells treated with compounds and [A] control is the absorbance of control cells. IC_50_ values are presented in Table 4.

## 4. Conclusions

The work developed in this analytical investigation allowed us to reach some conclusions about some new heterocyclic compounds as new chemotherapeutics, namely bioreductive prodrugs, which are activated under hypoxic conditions. To study the stability of some benzimidazole-4,7-dione and *N*-oxide benzimidazole-4,7-dione derivatives in a few solvent environments, UPLC analyses were executed.

The established analytical method allowed us to study the stability of compounds in some specific environments. The compounds were stable in all the solvents, as shown by the high half-lifetime. This information was very useful for our further biological studies with cancer cell lines, in which potential bioreductive prodrugs (such as the ones developed in this research work) were tested.

The biological study was conducted in 0.2% DMSO and it proved that compound **5a** was the most effective in inhibiting the growth of normoxic as well as hypoxic A549 cells. Compounds **6b** and **6a** were more potent to specifically inhibit the cell viability of hypoxic cancer cells while they were less effective in normoxic cells. Moreover, hypoxic/aerobic cytotoxicity coefficient of compound **6b** was 4.31 while for tirapazamine it was 4.61. This parameter classifies compound **6b** as a new bioreductive agents between mitomycin C (cytotoxic coefficient from 1 to 5) and 2-nitroimidazole (misonidazole–toxicity coefficient from 5 to 15) [15,29,30].

## Supplementary Materials

Supplementary materials can be accessed at: http://www.mdpi.com/1420-3049/19/1/400/s1.
