# Supplementary material for: Synthesis, Anticancer Activity and UPLC Analysis of the Stability of Some New Benzimidazole-4,7-dione Derivatives
Source: Molecules. 2013 Dec 31;19(1):400–13. doi: 10.3390/molecules19010400 (PMC6271476; doi:10.3390/molecules19010400)

Supplementary Materials for
Synthesis, Anticancer Activity and UPLC Analysis of the Stability of Some New Benzimidazole-4,7-dione Derivatives

2-(4-Chlorophenyl)-1*H*-benzimidazol-4,7-dione (5a)

IR (KBr) υ/cm^{-1}: 3615 (NH), 1690 (C=O), 1485 (C=N); ^{1}H-NMR (DMSO-d_6) δ: 14.5 (s, 1H, NH), 8.2 (d, 2H, CH, J = 7.9 Hz, 7.6 (d, 2H, CH, J = 2.0 Hz), 7.5 (d, 2H, CH, J = 2.0 Hz); ^{13}C-NMR (DMSO-d_6) δ: 180.0, 179.1, 150.2, 143.4, 142.8, 136.8, 135.7, 129.9, 129.3, 128.7, 127.9, ; MS *m/z* [M+1, M−1]: 259, 257.

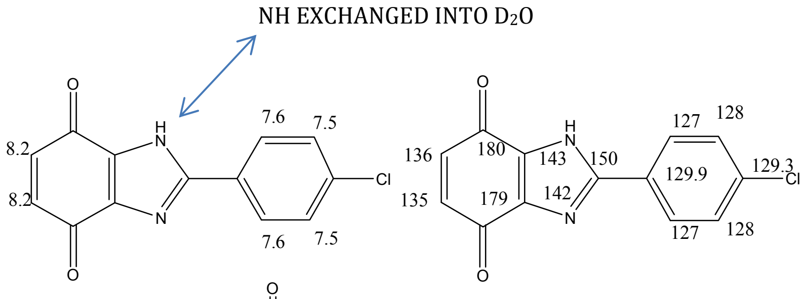

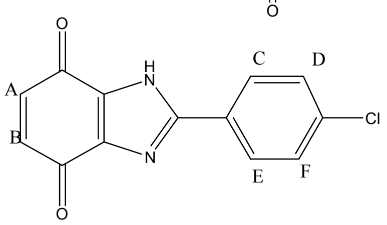

$J_{AB} = 7.9$; $J_{CE,DF} = 2.0$

2-(2-Nitrophenyl)-1*H*-benzimidazol-4,7-dione (5b)

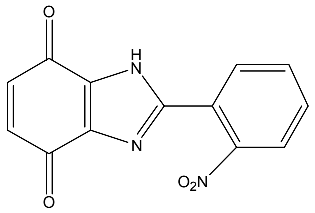

IR (KBr) υ/cm^{-1}: 3391 (NH), 1690 (C=O), 1526 (NO_2asym), 1347 (NO_2sym), 1481 (C=N).

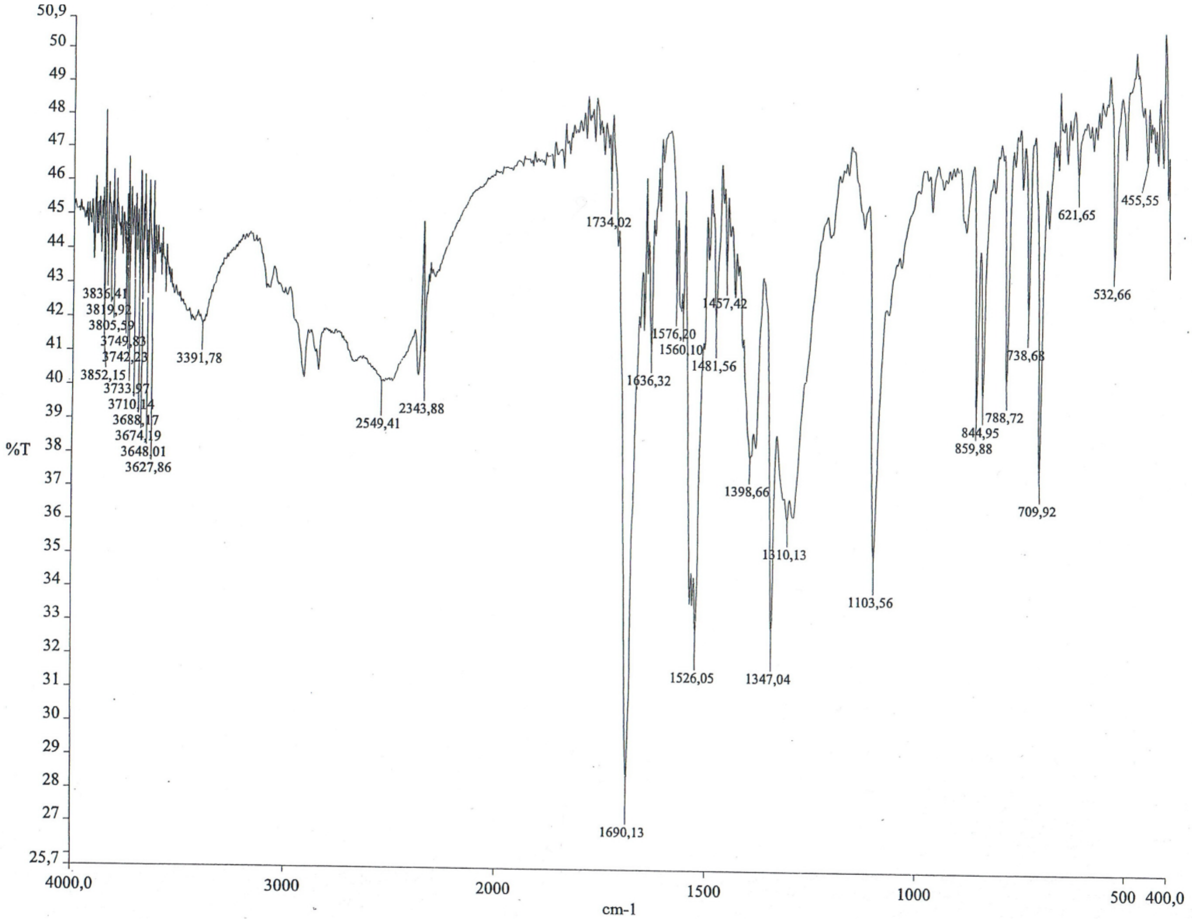

^{1}H-NMR (DMSO-d_6) δ: 9. 2 (s, 1H, NH), 8.1 (d, 2H, CH, J = 7.9 Hz), 7.8 (m, 4H, CH); 8.1 (d, 2H, CH, J = 7.9 Hz) = J_{AB}; NH signal exchanged with D_2O.

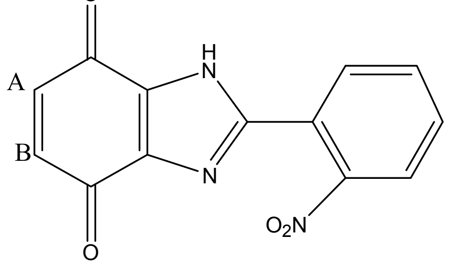

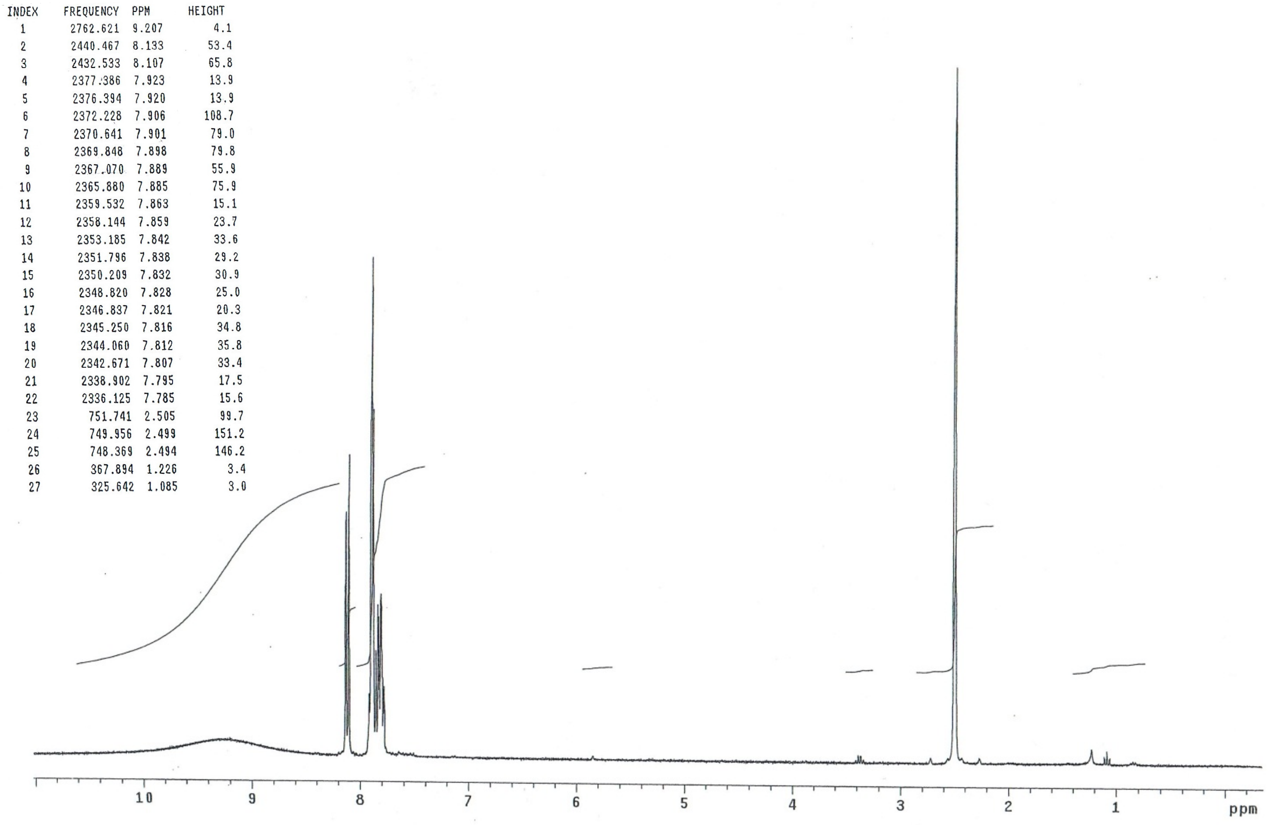

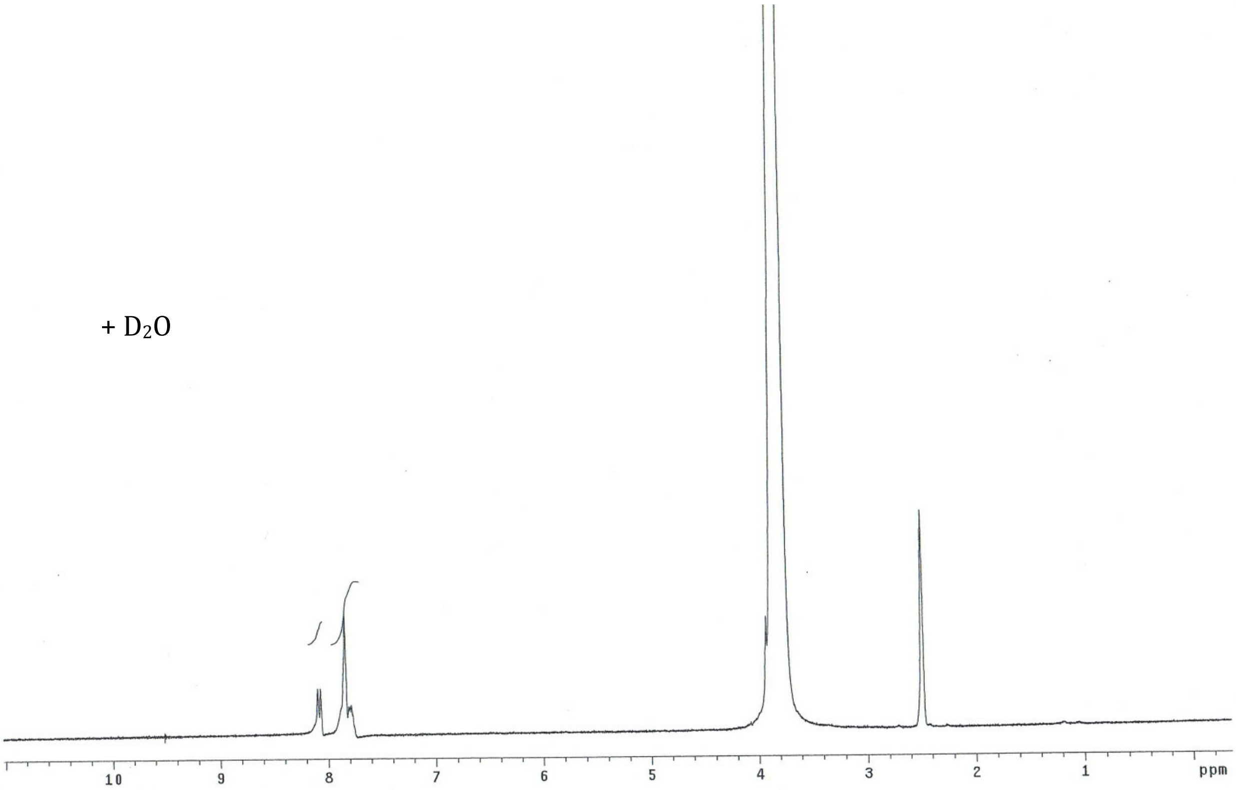

^{13}C-NMR (DMSO-d_6) δ: 181.2, 179.1, 148.7, 147.2, 146.7, 146.1, 142.9, 141.9, 133.1, 131.9, 128.1, 125.5, 124.6.

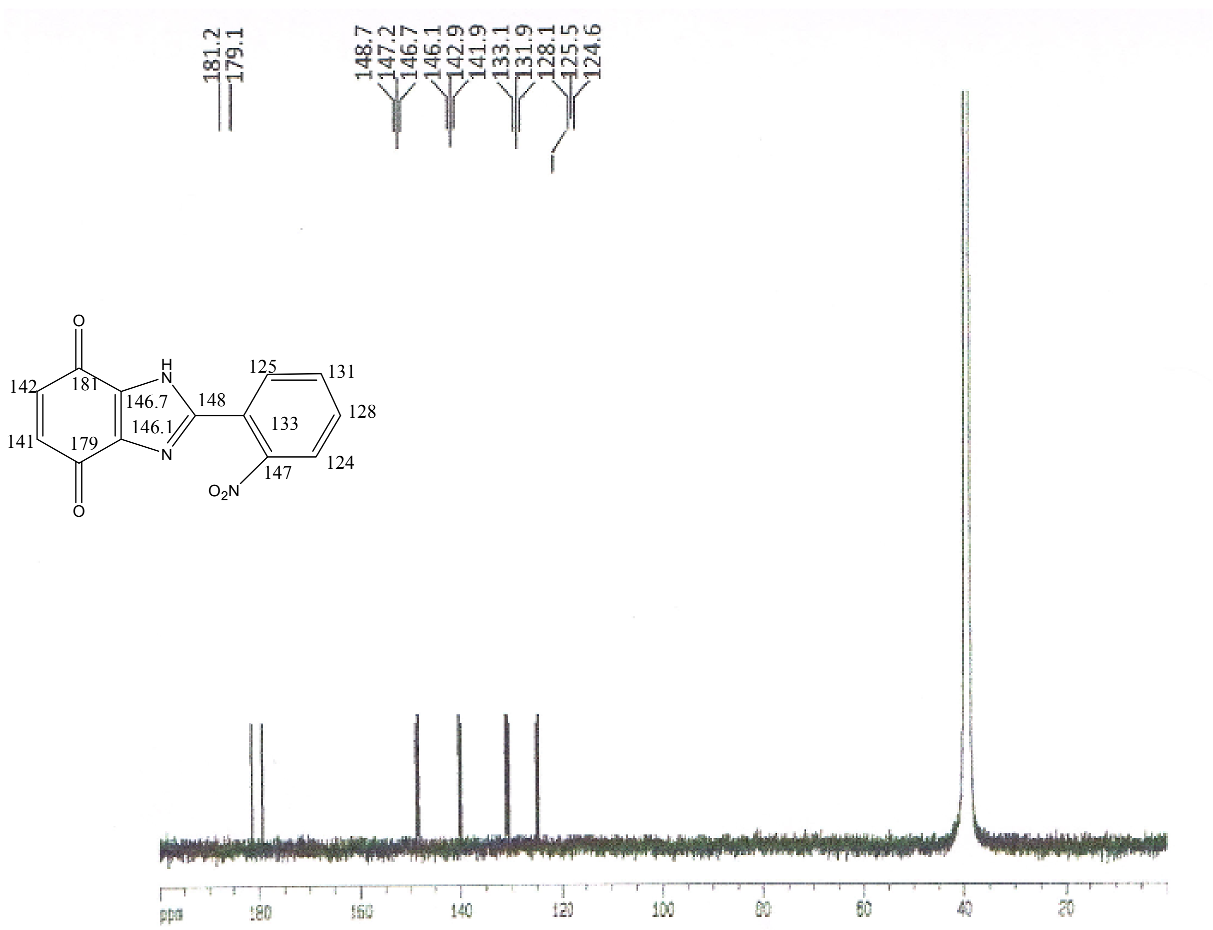

MS *m/z* [M+1, M−1]: 270, 268.

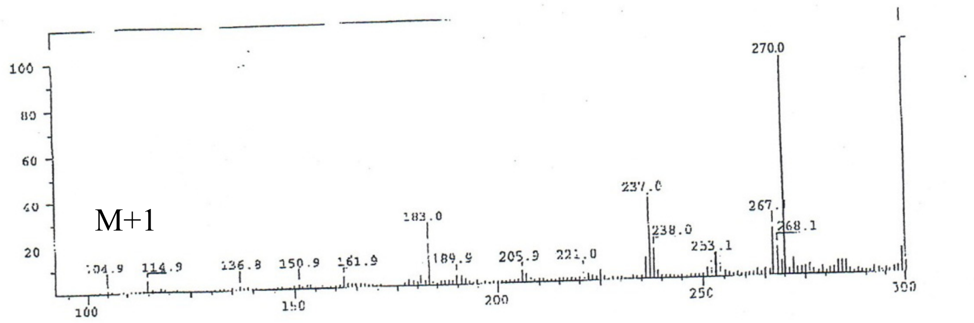

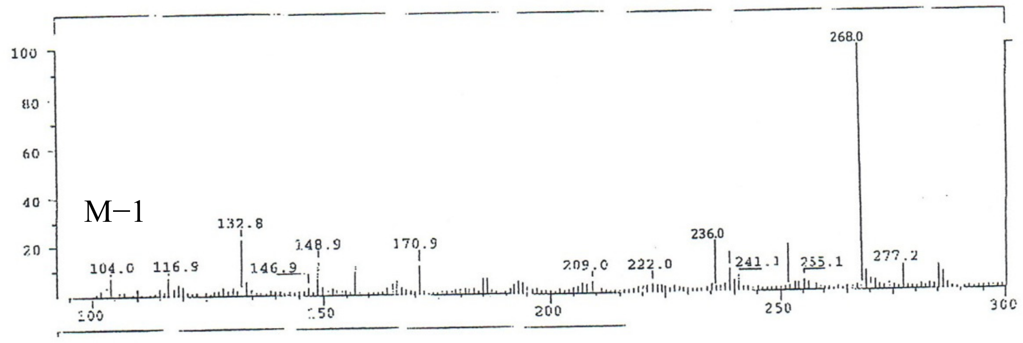

2-Benzo[1,3]dioxol-1*H*-benzimidazol-4,7-dione (5c)

IR (KBr) υ/cm^{-1}: 3324 (NH), 2960 (CH_2), 1701 (C=O), 1503 (C=N), 1264 (C-O-Csym), 1036 (C-O-Casym); ^{1}H-NMR (DMSO-d_6) δ: 4.0 (s, 1H, NH), 7.9 (d, 2H, CH, J = 1.98 Hz), 7.2 (d, 1H, CH, J = 7.9 Hz), 7.0 (d, 2H, CH, J = 8.0 Hz), 6.2 (s, 2H, CH_2); ^{13}C-NMR (DMSO-d_6) δ: 179.5, 179.0, 149.1, 148.6, 147.6, 142.1, 141.5, 141.3, 141.1, 129.1, 120.3, 115,1, 113.2, 91.

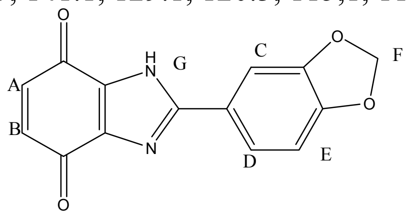

J_{AB} = 8.0, J_{CD} = 1.98, J_{DE} = 7.9

2-Naphthyl-1*H*-benzimidazol-4,7-dione (5d)

IR (KBr) υ/cm^{-1}: 3424 (NH), 3033 (ArH), 1677 (C=O); 1452 (C=N); ^{1}H-NMR (DMSO-d_6) δ: 13.0 (s, 1H, NH), 8.7 (s, 1H, CH) 8.3 (d, 2H, CH, J = 1.8 Hz), 8.1 (d, 2H, CH, J = 8.1Hz), 7.9 (d, 2H, CH, J = 1.8 Hz), 7.6 (d, 2H, CH, J = 1.8 Hz); ^{13}C-NMR (DMSO-d_6) δ: 179.9, 179.1, 150.2, 148.6, 148.5, 147.7, 147.3, 142.2, 141.2, 134.1, 133.8, 133.6, 128.5, 126.5, 124.3.

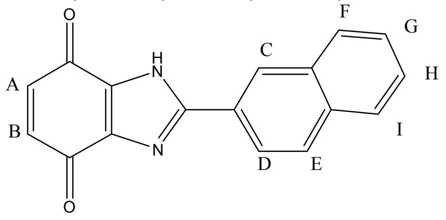

J_{AB} = 8.1; $J_{CD} = _{FH} =_{IG}$ = 1.8

2-(4-Chlorophenyl)-1*H*-benzimidazol-4,7-dione N-oxide (6a)

IR (KBr) υ/cm^{-1}: 3445 (NH), 1685 (C=O), 1661 (C=O), 1484 (C=N), 1282 (N-O); ^{1}H-NMR (DMSO-d_6) δ: 14.5 (s, 1H, NH), 8.1 (d, 2H, CH, J = 8.0 Hz), 7.6 (d, 2H, CH, J = 2.0 Hz), 7.5 (d, 2H, CH, J = 2.0 Hz), ^{13}C-NMR (DMSO-d_6) δ: 180.2, 179.3, 157.2, 143.1, 142.6, 136.5, 135.2, 129.8, 129.1, 128.6, 128.0; MS *m/z* [M+1, M−1]: 275, 273.

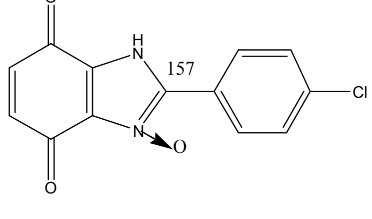

2-(2-nitrophenyl)-1*H*-benzimidazol-4,7-dione N-oxide (6b)

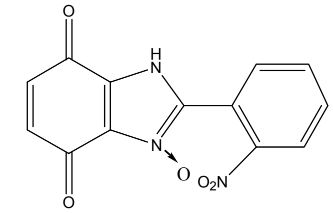

IR (KBr) υ/cm^{-1}: 3480 (NH), 1682 (C=O), 1541 (NO_2asym), 1347 (NO_2sym) 1434 (C=N), **1251** (**N-O**).

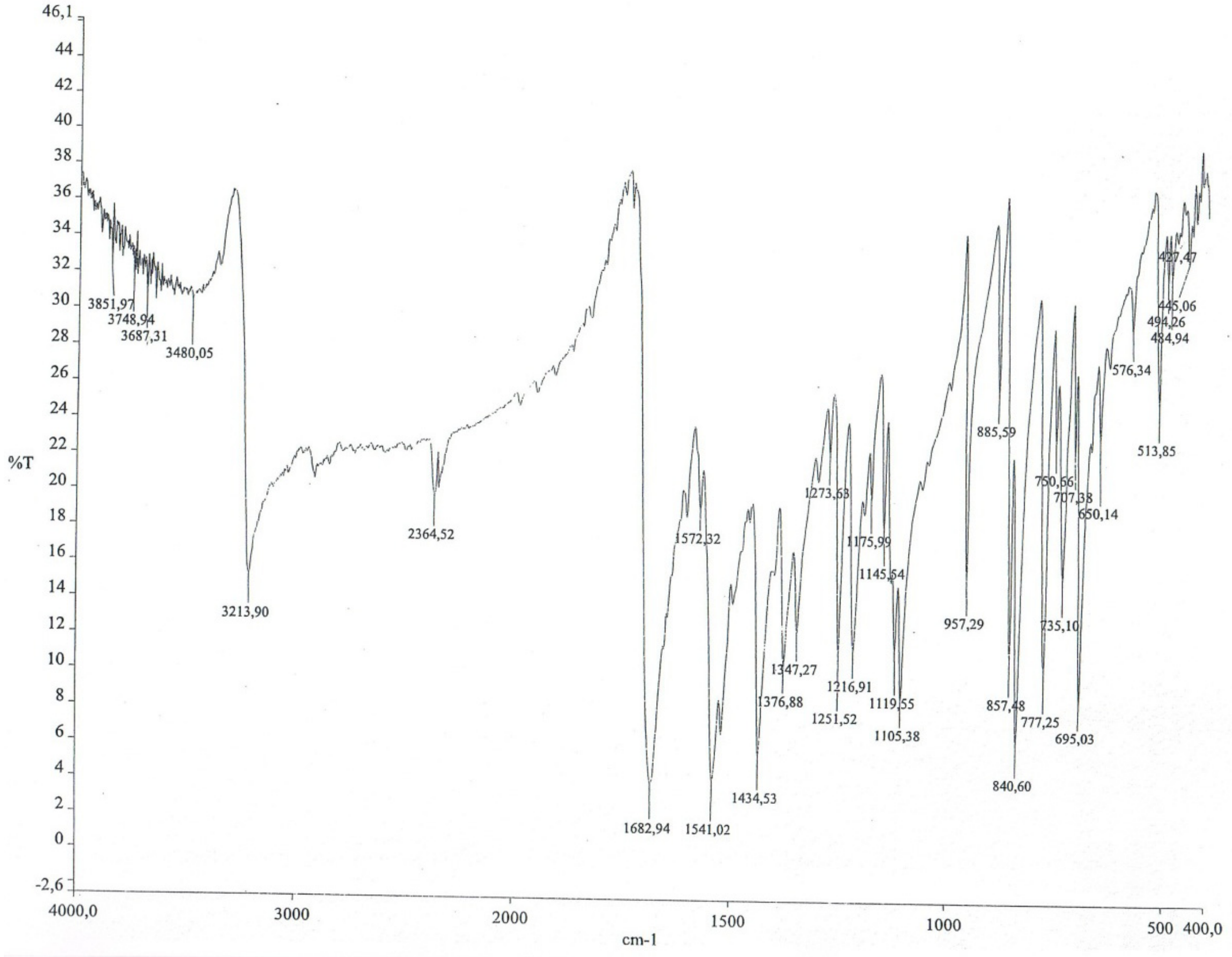

^{13}C-NMR (DMSO-d_6) δ: 180.2, 179.3, 157.7, 147.6, 146.2, 146.0, 143.0, 142.4, 133.5, 131.5, 128.4, 125.3, 124.1.

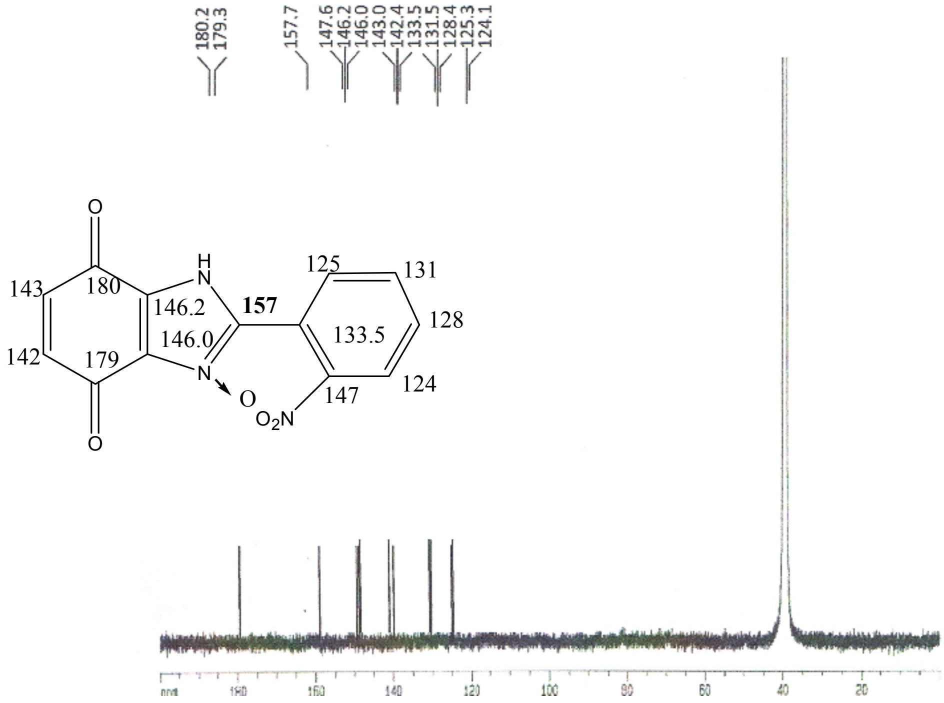

MS *m/z* [M+1, M−1]: 286, 284.

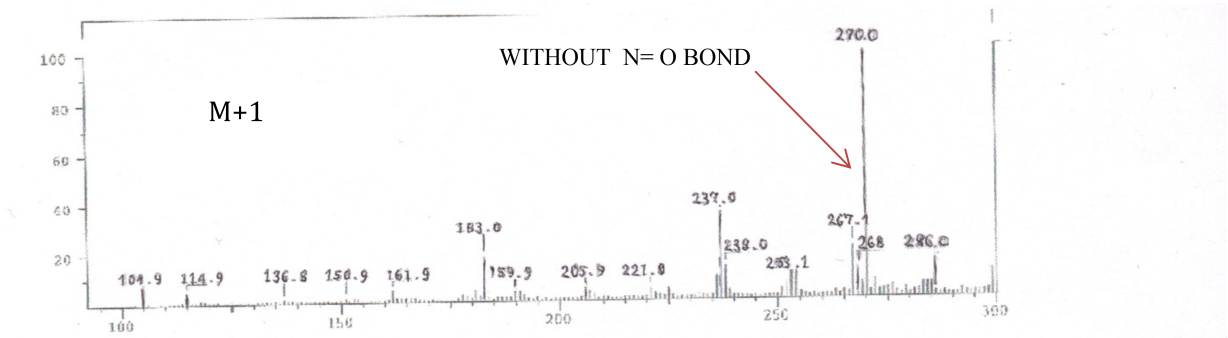

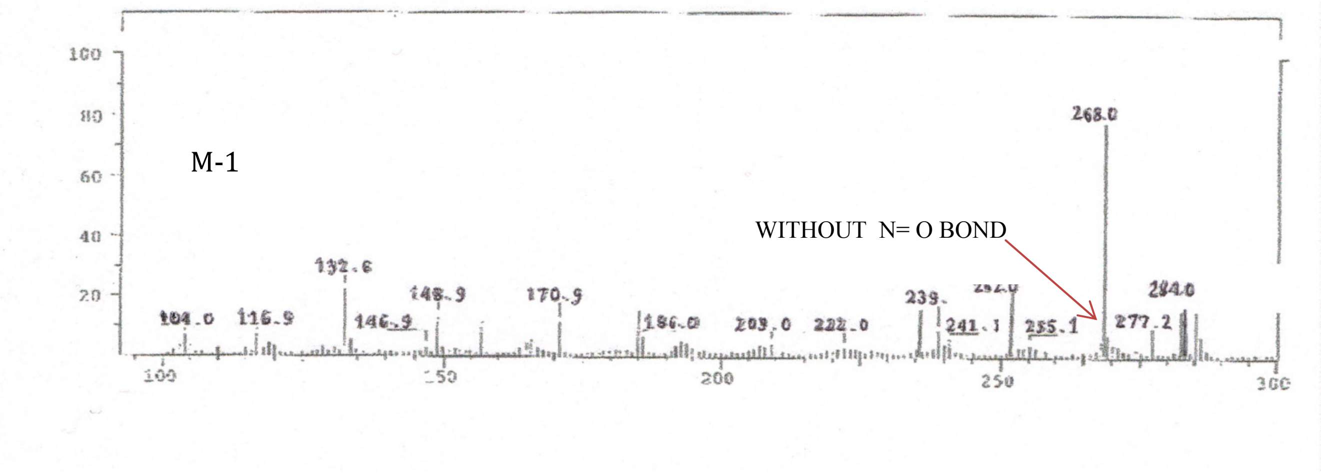

2-Benzo[1,3]dioxol-1*H*-benzimidazol-4,7-dione *N*-oxide (6c)

IR (KBr) υ/cm^{-1}: 3346 (NH), 2961 (CH_2), 1680 (C=O), 1470 (C=N), 1255 (C-O-Csym), 1045 (C-O-Casym), 1249 (N-O); ^{1}H-NMR (DMSO-d_6) δ: 10.0 (s,1H,NH), 7.8 (d, 2H, CH, J = 8.1 Hz), 7.7 (d, 1H, CH, J = 8.0 Hz), 7.6 (d, 2H, CH, J = 2.0 Hz), 6.0 (s, 2H, CH_2); ^{13}C-NMR (DMSO-d_6) δ: 18025, 179.8, 159.1, 148.2, 147.1, 142.0, 141.7, 141.5, 141.0, 129.3, 120.1, 115.5, 113.3, 91.2.

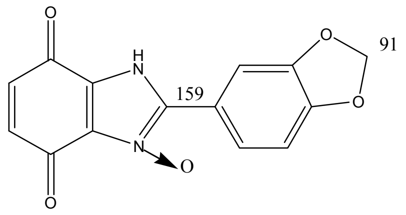

2-Naphthyl-1*H*-benzimidazol-4,7-dione *N*-oxide (6d)

IR (KBr) υ/cm^{-1}: 3300 (NH), 3031 (ArH), 1670 (C=O), 1465 (C=N), 1261 (N-O); ^{1}H-NMR (DMSO-d_6) δ: 13.1 (s, 1H, NH), 8.8 (s, 1H, CH), 8.3 (d, 2H, CH, J = 2.0 Hz) 8.0 (d, 2H, CH, J = 7.9 Hz), 7.6 (d, 2H, CH, J = 2.0 Hz), 7.4 (d, 2H, CH, J = 2.0 Hz); ^{13}C-NMR (DMSO-d_6) δ: 179.5, 179.2, 160.1, 147.7, 147.5, 142.6, 142.1, 141.0, 140.2, 134.3, 133.7, 133.5, 128.1, 126.2, 124.7.

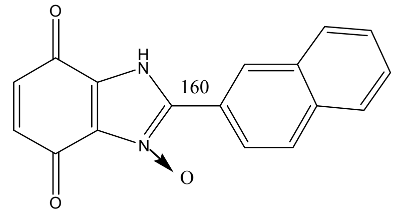

Figure S1. Chromatograms and UV spectrums of analysed compounds. **A** and **B**, Chromatogram and UV spectrum of **5b**; **C** and **D**, Chromatogram and UV spectrum of **6b**.

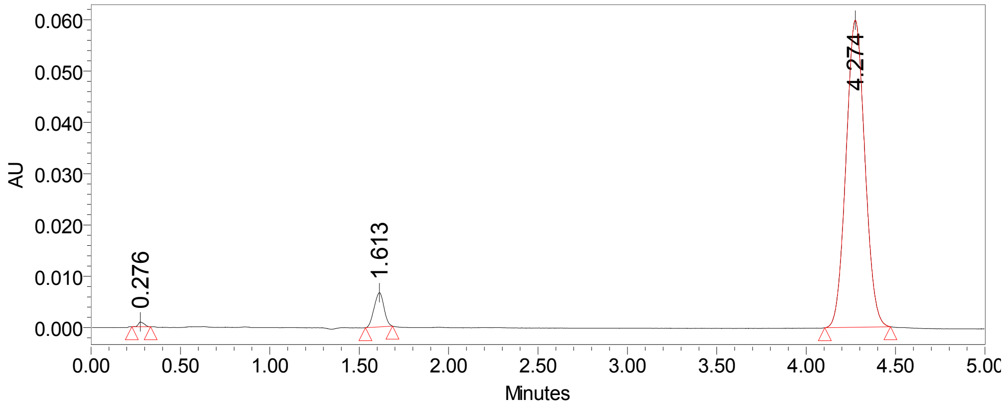

A

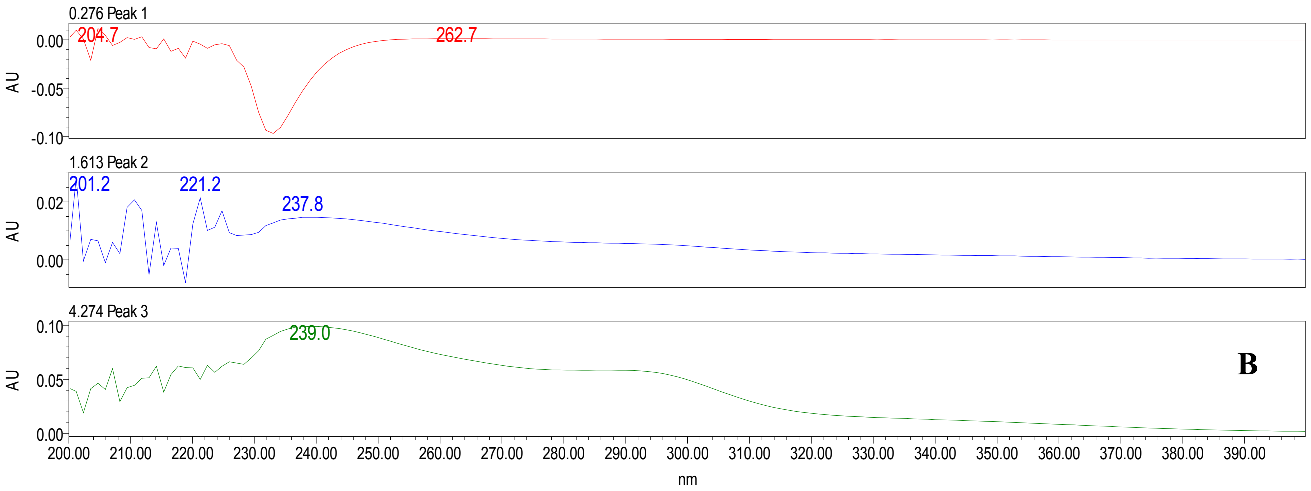

B

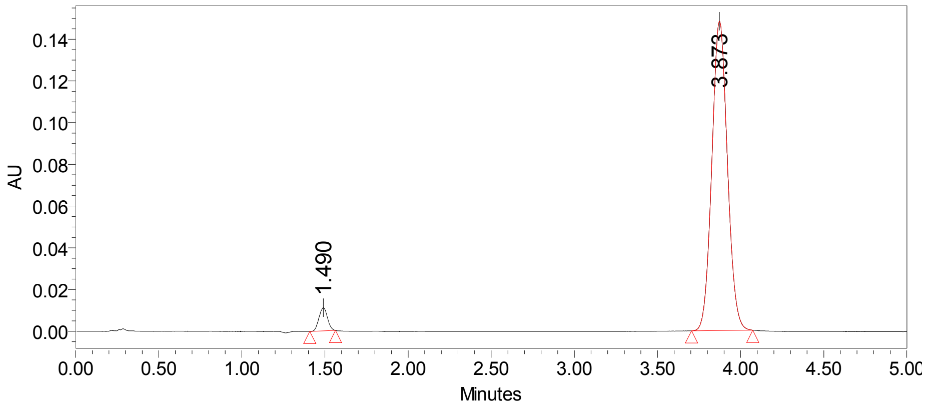

C

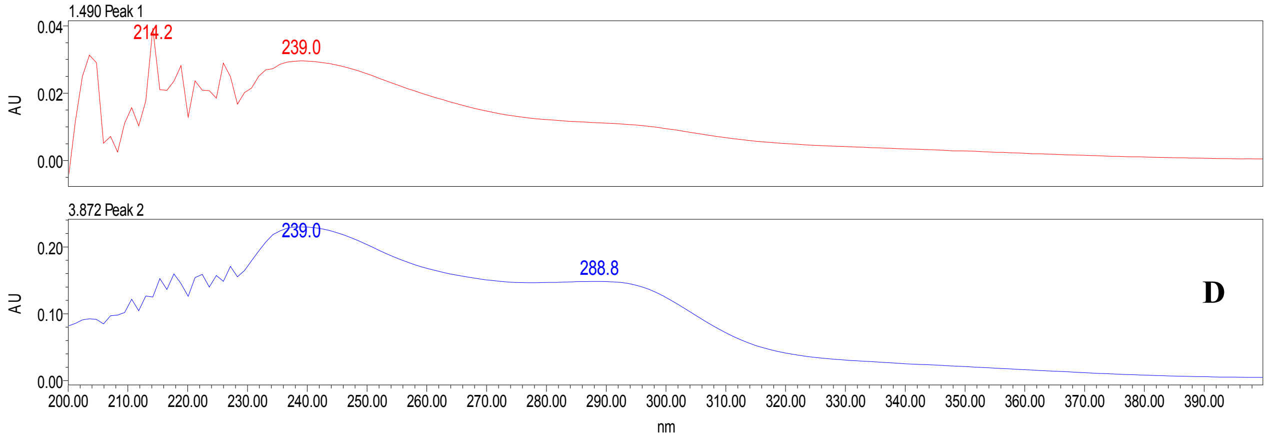

D

Table S1. Precision of the analytical method.

**Conc. (mg/mL) n = 3**	**Compound 5b**					
	25%	39%	52%	57%	78%	100%
$\bar{x}$	0.052	0.081	0.108	0.120	0.162	0.209
**S**	0.0006	0.0006	0.0010	0.0006	0.0012	0.0006
**μ**	0.052 ± 0.0012	0.081 ± 0.0012	0.108 ± 0.0020	0.120 ± 0.0012	0.162 ± 0.0023	0.209 ± 0.0012
**RSD (%)**	1.10	0.72	0.93	0.48	0.71	0.28
**Conc. (mg/mL) n = 3**	**Compound 6b**					
	25%	35%	51%	63%	84%	100%
$\bar{x}$	0.053	0.073	0.107	0.132	0.175	0.210
**S**	0.0006	0.0006	0.0006	0.0010	0.0006	0.0006
**μ**	0.053 ± 0.0012	0.073 ± 0.0012	0.107 ± 0.0012	0.132 ± 0.0020	0.175 ± 0.0012	0.210 ± 0.0012
**RSD (%)**	1.10	0.79	0.54	0.76	0.33	0.28

$\bar{x}$, arithmetic mean; s, standard deviation; μ, mean ± SD; RSD (%), relative standard deviation.

Table S2. Accuracy of the analytical method.

	Content of the determined substance **5b** in relation to the declared (mg/mL)							
	**25%**		**50%**		**75%**		**100%**	
**Samples**	**Real value**	**Determined value**	**Real value**	**Determined value**	**Real value**	**Determined value**	**Real value**	**Determined value**
**1**	0.05	0.05	0.104	0.104	0.157	0.157	0.209	0.209
**2**	0.055	0.055	0.111	0.111	0.166	0.166	0.222	0.221
**3**	0.026	0.025	0.052	0.053	0.078	0.078	0.104	0.104
	mean recovery (%) = 100.07; s (%) = 0.54; μ (%) = 100.07 ± 1.08; RSD (%) = 0.54							
	Content of the determined substance **6b** in relation to the declared (mg/mL)							
	**25%**		**50%**		**75%**		**100%**	
**Samples**	**Real value**	**Determined value**	**Real value**	**Determined value**	**Real value**	**Determined value**	**Real value**	**Determined value**
**1**	0.06	0.06	0.115	0.115	0.172	0.172	0.23	0.23
**2**	0.066	0.066	0.132	0.132	0.197	0.197	0.263	0.262
**3**	0.06	0.06	0.125	0.125	0.187	0.187	0.25	0.25
	mean recovery (%) = 98.99; s (%) = 0.82; μ (%) = 100.07 ± 1.64; RSD (%) = 0.83							

$\bar{x}$, arithmetic mean; s, standard deviation; μ, mean ± SD; RSD (%), relative standard deviation.

Figure S2. Cell viability of compounds **5a**–**b** and **6a**–**b** at normoxia and hypoxia conditions. Data is expressed as mean-SD, n = 3, 0—control, , T-tirapazamine.

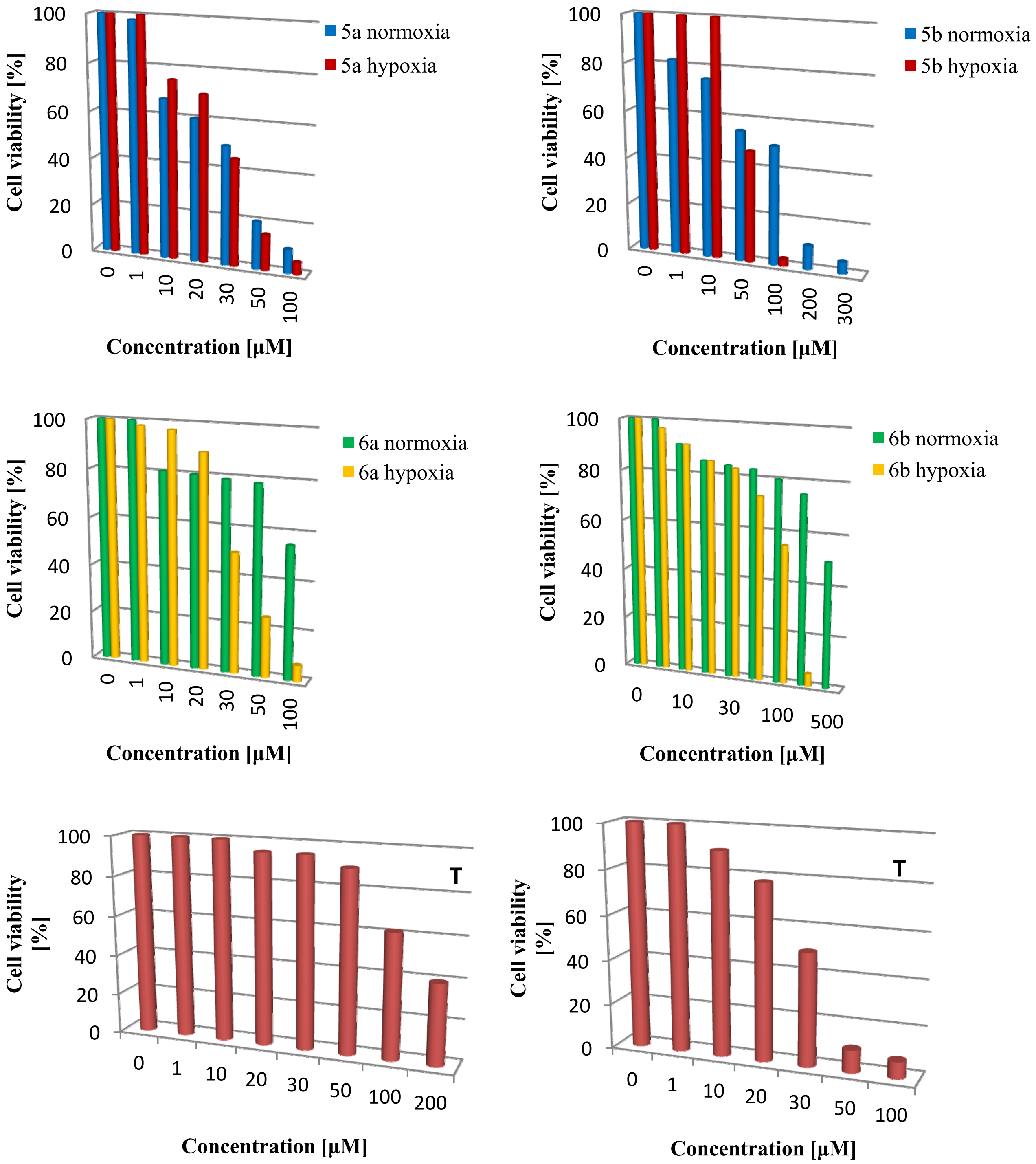

Supplement: Supplementary file 1 [file molecules-19-00400-s001.pdf]
